# Trial Protocol for Evaluating Platforms for Growing Microgreens in Hydroponic Conditions

**DOI:** 10.3390/foods11091327

**Published:** 2022-05-03

**Authors:** Paula Ioana Moraru, Teodor Rusu, Olimpia Smaranda Mintas

**Affiliations:** 1 Department of Technical and Soil Sciences, Faculty of Agriculture, University of Agricultural Sciences and Veterinary Medicine Cluj-Napoca, 400372 Cluj-Napoca, Romania; paulaioana.moraru@usamvcluj.ro; 2 Faculty of Environmental Protection, University of Oradea, 410087 Oradea, Romania; buzasiu@yahoo.com

**Keywords:** microgreens, hydroponic, trial protocol, production and quality paremeters

## Abstract

The hydroponic production of microgreens has potential to develop, at both an industrial, and a family level, due to the improved production platforms. The literature review found numerous studies which recommend procedures, parameters and best intervals for the development of microgreens. This paper aims to develop, based on the review of the literature, a set of procedures and parameters, included in a test protocol, for hydroponically cultivated microgreens. Procedures and parameters proposed to be included in the trial protocol for evaluating platforms for growing microgreens in hydroponic conditions are: (1) different determinations: in controlled settings (setting the optimal ranges) and in operational environments settings (weather conditions in the area/testing period); (2) procedures and parameters related to microgreen growth (obtaining the microgreens seedling, determining microgreen germination, measurements on the morphology of plants, microgreens harvesting); (3) microgreens production and quality (fresh biomass yield, dry matter content, water use efficiency, bioactive compound analysis, statistical analysis). Procedures and parameters proposed in the protocol will provide us with the evaluation information of the hydroponic platforms to ensure: number of growing days to reach desired size; yield per area, crop health, and secondary metabolite accumulation.

## 1. Introduction

Microgreens are young plants that are consumed at the seedling stage, which have a short production cycle (about 14 days) and require little space for growth [1]. Microgreens are emerging functional foods of the 21st century [2] that are gaining interest for their sustained nutraceutical properties and are an optimistic prospect for expanding especially for the consumption of the population in large urban areas and in terms of food security. Production of microgreens using hydroponic systems must be planned and controlled with care for controlling environmental factors in order to increase quality parameters [3,4,5,6]. This is in comparison to more conventional production methods using soil, considering all the controllable factors in hydroponic systems that have been shown to influence the accumulation of bioactive substances [7,8], the harvest timeframe [9,10,11], and the quality of the finished product [12,13,14]. Furthermore, the lack of a soil’s microbiome in hydroponic systems is also important to consider, as unsuccessful parameterization leaves the plants vulnerable to harmful spoilage by microorganisms [15,16,17].

However, the advantages of hydroponic platforms and the development of evaluation protocols can lead to a positive influence on the quality of microgreens with higher concentrations of active substances [18,19] and nutrients valuable for human health [20,21,22]. This is why it is necessary to standardize certain cultivation protocols to ensure their quality [23,24,25]. For instance, there is a wide range of environmental impact factors and variation in their relationships to downstream microgreens outputs, which means that there is no single prescription that will guarantee perfect results [26]. The literature review has demonstrated that there are optimal ranges within which one can begin the task of designing effective prescriptions for successful microgreen production [3,21,27].

The time from sowing to harvest is 7–21 days for microgreens [28], a period in which the control of vegetation factors is very important. Nutritional solution, temperature, and light regime have the most important role in seed germination [29,30] and development [31], while also summarizing the recent research on the many promising research trends in refining microgreen production to achieve optimal outputs along its phenological stages [32]. The nutritional solution, air, and water temperature, light regime, pH, electrical conductivity, dissolved oxygen, CO_2_ concentration, and relative humidity are all important factors which influence secondary metabolism from an incipient phase [33,34], which in the final stages increases both the perceived and actual value of the plants by contributing to human health and nutritional fortification [35,36].

Microgreen producers must integrate specific systematic hydroponic strategies to obtain high-quality microgreens and high quantity [37] and quality bioactive substances [38], while also avoiding the potential for spoilage and low-quality production [20,39] when moving too far beyond the noted parameter ranges [3].

Many authors in the literature review have noted that best practices have not been developed [40,41], which means that although there are many guidelines for producing microgreens, we do not have very clearly defined standards; this literature review has therefore gathered critical information regarding hydroponically grown microgreen production that can be used by researchers and producers to improve the protocol for testing platforms used to obtain microgreens [18,42].

Microgreens are currently considered among the five most profitable crops, along with mushrooms, ginseng, saffron and goji berries [43]. Therefore, developing species-specific growth media to support year-round production and to enhance valuable antioxidant components is affordable and of utmost importance for the microgreens industry [19,22,43]. It is particularly important that the fundamental research into ensuring the safety and quality of this new addition to healthy diets, microgreens, is carried out so that the produce industry can avoid some of the problems that have challenged the mature produce and sprout industries during the past several decades [44,45].

The paper aims to develop, based on the review of the literature, a set of procedures and parameters, included in a test protocol, for hydroponically cultivated microgreens in order to optimize the cultivation process and allow the harvest of the best possible products in any hydroponic installation. Pilot trials target research into microgreens, specifically, the influence of the crop environment and of the environment factors on the growth and development of plants under hydroponic conditions.

## 2. Scope and Approach

This review was conducted as part of the GoHydro project (https://gohydro.org, accessed on 18 April 2022). The objective of this activity was to develop a trial protocol for hydroponic platforms for obtaining microgreens. The parameters established in the protocol have been selected so as to provide the best information on the operation of the platform. Feedback from these trials will be used for the final validation of the analytics components of the GoHydro platform.

**Figure 1:**
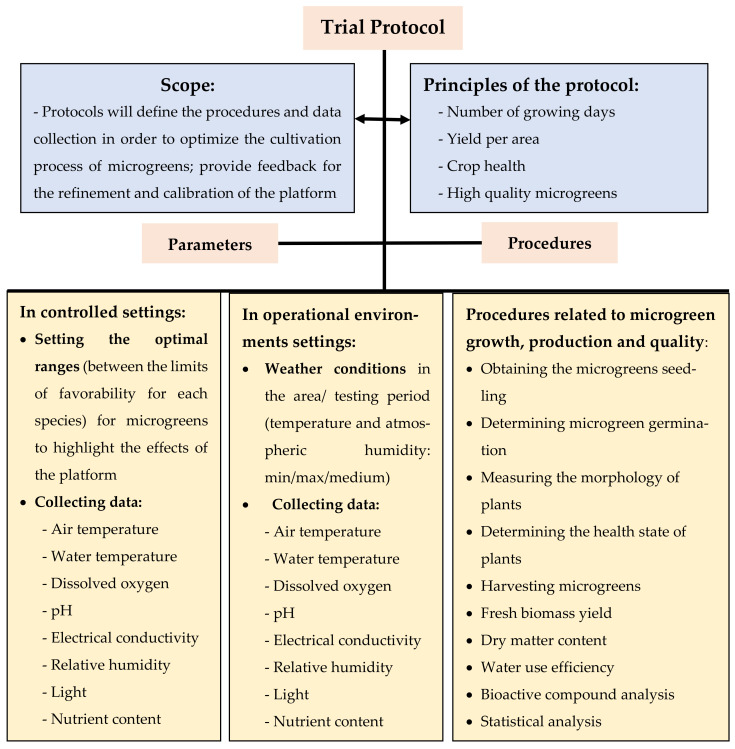
Scheme of procedures and parameters proposed for the trial protocol.

The procedures and parameters proposed to be included in the protocol are (Figure 1): (1) different determinations: in controlled settings (setting the optimal ranges), and in operational environments settings (weather conditions in the area/testing period); (2) procedures and parameters related to microgreen growth (obtaining the microgreens seedling, determining microgreen germination, measurements on the morphology of plants, microgreens harvesting); (3) microgreens production and quality (fresh biomass yield, dry matter content, water use efficiency, bioactive compound analysis, statistical analysis).

In the protocol, we consider that microgreen growth could be characterized by four main variables that are not necessarily correlated [46]: (1) number of growing days to reach desired size; (2) yield per area (for given number of seeds), (3) crop health (percentage of crops diseased); and (4) secondary metabolite accumulation (ascorbic acid, carotenoids, chlorophyll, etc.).

The literature review, carried out by us, with a focus on literature from the last 10 years, was conducted between November 2021 and April 2022, using the databases: Web of Sciences, Scopus, Science Direct, and Google Scholar. The established procedures and parameters are analyzed with the goal to highlight, within the tested platforms, in what way, different environment and nutritional factors (used as keywords in the review process) can influence the development of microgreens and can improve its production and quality.

## 3. Trial Protocol for Evaluating Platforms for Growing Microgreens

The trial protocol for evaluating platforms for growing microgreens in hydroponic conditions includes the procedures to be followed and the parameters considered useful for calibrating the platform.

Hydroponic GoHydro systems (https://gohydro.org, accessed on 18 April 2022). have specific characteristics, such as the layer of crop used (nutrient solution), type of irrigation (closed), method of irrigation (immersing), irrigation level (root level) [47]. Plants are cultivated in a substrate membrane, over which the nutrient solution passes periodically [48].

The high-capacity tank helps to maintain a constant pH. The color of the tank must be white on the outside to maintain a constant temperature of the nutrient solution, and it is not affected by solar radiation [49]. The water pump is in the tank, and the nutrient solution reaches the surface of containers through a pipe system. The pump recirculates the whole solution within 30 min of a fertilization regime, and the result is the mixing of the solution in the system [50].

Microgreens can germinate and grow without any fertilizer application, up to the capacity of the specific seed’s capacity [39]. However, providing mineral nutrients to microgreens will increase yields and secondary metabolite concentration [51].

### 3.1. Setting the Optimal Ranges, in Controlled Settings

Setting the optimal ranges for microgreens, in controlled settings, between the limits of favorability for each species, aims to highlight the effects of the hydroponic platform [3]. As reported in the literature, special attention must be addressed to the choice of growth medium, which represents one of the key factors in the production process and could influence microgreens yield and quality [52]. Parameters defined and optimal ranges for different species of microgreens continuously monitored and controlled are presented in Table 1 [3,19]. The spectral output of the lighting system must be quantified using a spectrometer, at various points of growth of the trays of the platform [53].

The vegetation chamber is controlled by a system operated through a software program. The environmental factors (temperature, humidity, light) are controlled and monitored throughout the entire experimental period for the controlled experiments. Thus, for example, in the case of basil, the environment factors from the vegetation chamber shall be set as follows: temperature 21 ± 2 Day/17 Night; humidity 65 ± 5%, additional light by lamps of 400 W, photoperiodism: 06:30–21:30 (15 h), automatic airing at ±2 °C, compared to the programmed temperature. Table 2 presents measurement units, methods and possible equipment to be used.

The nutrient solution shall be changed every 10 days (at 8 o’clock in the morning) in order to satisfy the need for macro- and micro-elements [49]. After each change, the systems and all the devices used shall be disinfected [54]. The nutrient solution shall be monitored daily and manipulated in order to be maintained at the best parameters for the development of plants [55]. The level of the nutrient solution must be kept constant [19].

Measurements of the oxygen dissolved into water shall be made daily [55,56]. These measurements shall record the quantity of oxygen dissolved into water, the temperature of the solution, the date, the time and the temperature from the atmosphere.

Artificial lighting shall be measured on all the experimental surface, in different points [57], both from the point of view of intensity, and from the point of view of the quality of light. Light intensity shall be measured with a digital device which determines the number of photons relatively to surface and time (μmol m^−2^ s^−1^). The light spectrum shall be determined by using a spectrometer. These measurements shall be made above each tray [58] and at differences of 10, 20, 30 and 40 cm above their canopy.

In the case of measurements related to water losses, the crop trays shall be measured daily by using the digital scales [59].

### 3.2. Weather Conditions in the Area/Testing Period

In order to determine whether the weather conditions in the area/testing period have an influence on the operation of the hydroponic platform, important atmospheric data will be recorded daily [46]. The growing medium plays a very important role in determining the microgreens’ yield and quality [52], and the sustainability of the production process. The determined parameters will be: temperature min/max/medium (°C); atmospheric humidity min/max/mean (%).

### 3.3. Procedures and Parameters Related to Microgreen Growth

#### 3.3.1. Obtaining the Microgreens Seedling

In the case of hydroponic crops, the production of the seedling is essential in order to obtain uniform and quality microgreens. Varieties with rapid seed germination and not requiring low temperature treatments to stimulate it are preferred (lettuce may require precooling). This is preferable so as not to have an additional factor influencing the results.

The seeds are in seminal rest until the best medium allows for germination. The cultivation of microgreens requires an ample supply of neutral to slightly acidic water [44,60]. Seeds of some varieties are soaked overnight to enhance germination.

The crop sublayer, humidity, temperature and light regime have the most important role for the seed germination. The germination of microgreens seeds will be carried out in darkness at 20–24 °C (depending on the species) and 100% relative humidity [3]. For basil seeds, germination occurred in a climatic chamber in the dark at 24 °C for 3 days [25,61]. After approximately 3 days, the plants are exposed to light and watered daily until the first set of true leaves begin to emerge.

There are three distinctive phases in the process of seed germination [49]: water soaking, reinitiating metabolic activities from the seed, appearance of the radicle and its elongation.

Among common substrates used for the microgreens production, peat-based media are the most utilized, followed by coconut coir and several synthetic media [25]. Recently, natural fiber-based media—such as jute, cotton, cellulose, etc.—have gained increasing popularity since they could represent a sustainable alternative [43,62].

The seeds are placed directly in the sterile sublayer. Bulgari et al. (2021) [25] investigate the influence of three growing media (vermiculite, coconut fiber, and jute fabric) on yield and quality parameters of two basil varieties (green basil—*Ocimum basilicum* L., red basil—*Ocimum basilicum var. Purpurecsens*) and rocket (*Eruca sativa* Mill.) as microgreens. The results showed that the choice of the substrate significantly affected the yield, the dry matter percentage, and the nitrate concentration of microgreens, while the other qualitative parameters were most influenced by the species.

Seeds may require sterilization. Seed contamination is a well-known problem in the microgreens industry [63]. If seeds are contaminated, pathogens can become internalized from the beginning of the growing process and once incorporated are very difficult to remove [64]. During seed germination, the seed releases a mixture of carbohydrates and peptides that can attract surrounding bacteria in the rhizosphere [44,65]. It is recommended that the saturation with oxygen of the nutrient solution be maintained above 6.5 mg L^−1^, in order to eliminate the risk of the appearance of pathogens and for an optimal development of the root system [49].

Recently, studies have demonstrated that microgreen growing systems, especially hydroponic systems, are vulnerable to pathogen proliferation when seeds are contaminated, highlighting the importance of seed sanitation [44]. Some examples are summarized in Table 3.

Sanitization of the harvested product is not likely to be an effective control strategy [44]. Once contaminated, it is almost impossible to eliminate pathogens from living plant tissues [44,63]. Microgreens are very delicate and can be easily damaged by harsh sanitizing treatments [69].

Seeds should receive precautionary sanitary treatments for eliminating pathogenic bacteria such as those recommended for sprouts production by the U.S. Food and Drug Administration [70]. Tavan et al. (2021) [71], proposes that Tuscan black kale (*Brassica oleracea var. acephala*) seeds be first sterilized by soaking for 2 min in 80% ethanol, rinsed twice with distilled water, and then oven-dried at 45 °C for 40 min.

A textile material will be placed over the seeds to stop the light. After moistening and the beginning of the germination process (3 days), the textile material shall be removed for growing seedlings [72].

Bulgari et al. (2017) [73] recommend in the case of hydroponic cultivated basil, on polystyrene cell trays filled with vermiculite, a crop density of approximately 21,700 plants m^−2^ (about eight plants per cell).

To determine the density, depending on the species of microgreens, the amount of seeds can be calculated according to the size and shape of the trays using Microgreens Seed Density Calculator [74].

Seeding density impacts microgreens yield [24,75]; as the seeding density increases, the weight per individual plant decreases due to competition among seedlings, while the total yield increases from the increased number of seedlings in each area, up to an equilibrium production capacity.

#### 3.3.2. Determining Microgreen Germination

The proposed parameters for testing are as follows:

Germination Energy (GE, %)—is the speed at which the germination process is initiated in a seed placed under germination conditions. The percentage of pure seeds normally germinated in the period of 1/3 to ½ (usually 3–4 days) of the time established for the determination of the germination capacity is expressed [76].

Germination Capacity (GC, %)—is the capacity of the seeds to germinate, in a limited number of days, established for each species (7–10 days) [77]. It is expressed as a percentage of the number of pure seeds germinated.

Germination Index (GI) [78,79,80]—determining the germination index shall be assessed when this vegetation phase is finalized; the germination of seeds is considered complete when the petiole has reached at least the minimum same dimension as the dimension of the seed; measurements shall be made daily, until the 10th day, at the same hour.

GI = [Number of germinated seeds (n1)/Days of first count] + [Number of germinated seeds/Days of second count (n2)] + … + [Number of germinated seeds/Days of last or final count (n10)](1)

In the GI, maximum weight is given to the seeds germinated on the first day and less to those germinated later on. The lowest weight would be for seeds germinated on the 10th day. Therefore, the GI emphasizes on both the percentage of germination and its speed. A higher GI value denotes a higher percentage and rate of germination [79].

#### 3.3.3. Measurements on the Morphology of Plants

The determination is performed in the juvenile vegetative phase before harvesting the microgreens. The surface of the leaves (leaves area) will be determined with a planimeter on 10 plants per tray [73]. Ten representative plants for each tray of the platform will be harvested on the diagonals of the tray. Another possibility is to calculate the Leaf Area Index (LAI) by employing the formula of Fang et al. (2019) [81].

(2) $$\mathrm{LAI}\;=\frac{\mathrm{Leaf}\;\mathrm{area}\;\mathrm{per}\;\mathrm{plant}\;\left({\mathrm{cm}}^{2}\right)\;}{\mathrm{Land}\;\mathrm{area}\;\mathrm{occupied}\;\mathrm{by}\;a\;\mathrm{plant}\;\left({\mathrm{cm}}^{2}\right)\;}$$

#### 3.3.4. Determining the Health State of Plants

Due to the short crop time for microgreens, there are few severe pests or physiological disorders [82]. The most significant disease in microgreens production is damping off in recently germinated seedlings. Seeds can be sterilized prior to planting to minimize disease incidences.

Determining the health state of plants shall be achieved through the continuous monitoring of all the symptoms that appear [83]. The health state of plants shall be noted in ascending order with grades from 1 to 9, with the maximum grade corresponding to a perfect health state [84]. The results shall be presented as average values of repetitions, graphically represented compared to time.

We consider it appropriate to assess the average intensity of the disease attack using the FAO grades (Table 4; using grades 1 to 9) [84].

Next, we can calculate the degree of attack that represents the expression of the influence and severity of microgreens health.

The degree of attack (DA, %) is calculated according to the relation [84]:

DA, % = F, % × I, %/100(3)

where:

F, %—attack frequency of a phytopathogen;
I, %—attack intensity of a phytopathogen.

F, % = N × 100/Nt(4)

N = number of plants (organs) attacked.
Nt = total number of plants (organs) observed (controlled).

I, % = Σ (i × f)/n(5)

i = percentage of grade awarded.
f = number of plants (organs) marked with the respective note.
n = total number of attacked plants (organs) analyzed.

Recent studies on artificial lighting systems have shown that the quality of light and light spectrum can influence plants significantly [3]. There are multiples ways that this can have an influence; for example, it can influence plant health, increasing the concentration of active substances and thus improving the quality and improving the efficiency of use and marketing of microgreens (Table 5).

#### 3.3.5. Microgreens Harvesting

Microgreens are harvested, for analysis, when over 50% of them are at their best time for harvest. Microgreens are ready for harvest when they reach the first true leaf stage, usually at about 2 inches (5.08 cm) tall [28,90]. The recommended maximum height limit is 6 cm [91]. The time from seeding to harvest can vary greatly by crop from 7 to 21 days [28], but is typically around 14 days [63]. The use of seedling height as a harvesting index can be recommended, as it can be determined easily [91]. However, leaf area can also be used as a harvesting stage index [91]. As different parts of the plants, such as seeds, cotyledons and leaves, may have different health-promoting properties, the ideal time of consumption in order to benefit their phytochemicals varies [92], which shows the importance of determining antioxidant activity at different stages. No formal studies in the literature were found about how harvest age affects the shelf life of microgreens [44].

Production in small trays will likely require harvesting with scissors [28]. The majority of vegetable varieties grown as microgreens are ready for harvest in about 2 weeks. Pannico et al. (2020) [93], proposes for lettuce microgreens harvesting at 16 days after sowing, upon the appearance of the first two true leaves. They are weighed to determine the fresh vegetable mass, then they are dried (lyophilization) in the oven at 70 °C for 3 days [71], or 4 days [72], to constant weight [94]. Lyophilization is considered to be the best dehydration method for both storage and sample pre-treatment, since it does not cause thermal degradation of carotenoids [95]. Drying the vegetable material is an important process when it comes to a correct characterization of plants and the active substances accumulated by them. The dry matter shall be recorded next for each sample and then it shall be chemically analyzed.

Time of the day for harvesting may have significant implications for the bioactive composition [96] and shelf-life of microgreens [97]. Noichinda et al. (2007) [98] propose that the microgreens be harvested in the morning, so as to avoid exposure to light, opening of the stomata and possible tendencies to lose the weight of the preserved samples.

Careful harvesting is required and quick cooling removes the vital heat and suppresses the rate of respiration, spoilage and senescence [70]. Samples collected from the platform will be stored at −20 °C until analyzed [99].

Current dip/wash and drying procedures significantly reduce the quality of the microgreens since microgreens are very delicate [44]. Improved wash/drying technologies are necessary to provide ready-to-eat microgreens with better quality and longer shelf life [100]. The post-harvest wash step can be avoided when the microgreens are grown under controlled settings to minimize the microbial contamination [27,100]. Microgreens crops usually are grown indoors. Thus, the materials used for propagation can be easily decontaminated to maintain compliance with food safety regulations.

### 3.4. Microgreens Production and Quality

#### 3.4.1. Fresh Biomass Yield

All the microgreens within each tray will be cut right above the substrate level (cutting them at the base, excluding the substrate) and collected to determine Fresh Weight (FW, kg m^−2^) [25].

#### 3.4.2. Dry Matter Content

Dry Weight (DW, g m^−2^) will be measured on an analytical balance following lyophilization until a constant weight was reached. Each sample shall be dried in an oven at 70 °C during 3 days [71] until constant weight is reached and DW shall be recorded (at 75 °C, 4 days) [25]. The dry samples will be finely ground to be utilized for chemical analysis.

#### 3.4.3. Water Use Efficiency

Water Use Efficiency (WUE) can be an important indicator of the efficiency of the hydroponic platform. It is calculated based on total harvested biomass [71]:

WUE = TFW/ΣW(6)

where:

TFW—total harvested biomass, g.
ΣW—total water added to each growing container of hydroponic platform.

#### 3.4.4. Bioactive Compound Analysis

The most important bioactive compounds in microgreens include vitamins (vitamin C), minerals (copper—Cu, zinc—Zn, and selenium—Se), and phytochemicals (e.g., carotenoids and phenolic) [21].

Vitamin C, also known as ascorbic acid, is a potent antioxidant and is essential for a variety of biological functions [101], such as wound healing, collagen synthesis, and immune system regulation [102]. As microgreens are usually consumed fresh, Vitamin C can be largely retained without cooking [103]. For the total ascorbic acid (TAA, g kg^−1^) analysis, a UV-Vis spectrophotometer will be used [92].

Several trace minerals, i.e., Cu, Zn and Se, as cofactors or components of antioxidant enzyme, play an essential role in the endogenous antioxidant defense system of the human body, and are therefore referred to as antioxidant minerals [104]. The content of chemical elements (Ca, Mg, Na, K, Mn, Fe, Zn, Cu, Se) (g kg^−1^) and volatile oils (mg L^−1^), shall be determined by specific HPLC methodology (chromatographic) [105].

Phytochemicals (e.g., carotenoids and phenolics) are found in significant amounts in microgreens [22]. Carotenoids possess antioxidant activity and play important physiological roles in the human body [106]. Phenolic compounds are the most abundant secondary metabolites of plants ranging from small molecules, e.g., phenolic acids, to flavonoids with multiple rings, and to highly polymerized compounds, e.g., tannins [107]. Phenolics are antioxidants for plants to repair damage caused by free radicals and have shown many health benefits for humans [107].

Bioactive compound: carotenoids (µg mL^−1^) and total polyphenols (µg mL^−1^) will be analyzed by HPLC methodology [108].

#### 3.4.5. Statistical Analysis

All data will be analyzed for differences using SPSS software and will be presented as average ± SE (standard error). Average values must be separated by LSD test (*p* < 0.05). The data will be analyzed in combination and compared to obtain significance and establish optimal environmental conditions, which must be provided by the hydroponic platform [46]. Data collection will be carried out in three repetitions. The statistical processing through the analysis of the variance will thus highlight the differences that may exist between repetitions. This ensures the accuracy of the data, real feedback, and the possibility to improve the accuracy of the hydroponic platform.

## 4. Conclusions

One of the major limitations of the expansion of microgreen consumption is the rapid deterioration of their quality, which occurs immediately after harvest, thus limiting their marketing. From this point of view, the test protocol must provide a suitable platform for the hydroponic production of microgreens on different substrates, indoors, representing a sustainable alternative to conventional agriculture.

Many studies have shown that the variation in the content of bioactive compounds in microgreens is based on several factors, such as genetic material (species), cultivation conditions and light parameters (spectral quality and intensity), but also other variables (including nutrition/biofortification and growth medium) have implications for shaping the nutritional and phytochemical composition of microgreens. Despite the short growing cycle, special attention should be paid in the testing protocol to establishing growth media for microgreens, which is one of the most important factors in the production process that influences the quality of microgreens and highlights the characteristics of the hydroponic platform.

Feedback from these trials will be used for the final validation of the analytics components of the hydroponic platform.

## Figures and Tables

**Table 1:** Parameters defined for different species of microgreens continuously monitored and controlled.

No.	Parameter	Unit of Measurement	Average Value of Parameters for Example Species ***		
			Basil	Lettuce	Brussels Sprouts
1	Light	W	400	400	400
1.1	Photoperiodicity	h	06:30–21:30 (15 h) (10–20 h)	07:00–20:00 (12 h)	07:00–20:00 (12 h)
1.2	Light intensity	μmolm^−2^s^−1^	300 (200–400)	500	300 ± 15
1.3	Color spectrum	nm	440–460 (260–780)	440–460	400–700
1.4	Distance from light	cm	150—Lamps HPS * 40—Lamps LED *	150—Lamps HPS 40—Lamps LED	150—Lamps HPS 40—Lamps LED
2	Ambient air temperature	°C	21 ± 2 Day/17 Night	20 ± 2	24 Day/18 Night ± 2
3	Relative humidity	%	65 ± 5 (50–60)	80 ± 5	70/80% ± 5
4	Nutrient concentration	N-P-K: 3-2-3 (%)	changed every 10 days **	changed every 10 days	changed every 10 days
5	pH	pH units	6.8 ± 0.4	6.3 ± 0.4	6.0 ± 0.2
6	Electrical conductivity	mS	1.2 ± 0.2	1.8 ± 0.2	1.8 ± 0.2
7	Dissolved oxygen	mgL^−1^	6.5	6	6
8	Solution temperature	°C	20 ± 2	18 ± 2	20 ± 2

Note: * HPS-High Pressure Sodium; LED-Light emitting diodes. ** 8 o’clock in the morning; *** monitor daily at 8 o’clock in the morning in 3 repetitions.

**Table 2:** Recommended measurement methods and equipment.

No.	Parameter	Unit of Measurement	Methods	Equipment for Measuring (Example)
1	Light	W	HPS/LED	Parameter specific
1.1	Photoperiodicity	h	Soft setting	Clock
1.2	Light intensity	μmolm^−2^s^−1^	Number of photons	Digital device (Luxmeter, spectroradiometer)
1.3	Color spectrum	nm	Light spectrum	Spectrometer
1.4	Distance from light	cm	Adjustment	Ruler
2	Ambient temperature	°C	Temperature sensor	Temperature sensor
3	Humidity	%	Relative humidity	Hygrometer sensor
4	Nutrient	N-P-K: 3-2-3 (%)	Type of solution	Standard
5	pH	pH units	Solution reaction	Laboratory pH meter
6	Electrical conductivity	mS	Electrical conductivity in water	Digital electrical conductivity measurement water conductivity sensor
7	Dissolved oxygen	mgL^−1^	Oxygen level as % of Saturation	Oxygenometer
8	Solution temperature	°C	Temperature sensor	TMCx-HD Water Temperature Sensor

**Table 3:** Studies showing the possibility of microgreen contamination.

No.	Reference	Investigation Context	Results
1	Xiao et al., 2015 [66]	*Escherichia coli* O157:H7 were able to survive and proliferate significantly on radish microgreens in both soil-substitute and hydroponic production systems, with higher populations reported in the hydroponic production system.	The results showed that contaminated seeds led to systematic contamination of whole plants, including both edible and inedible parts, and seed coats remained the focal point of *Escherichia coli* O157:H7 survival and growth throughout the period of microgreen production.
2	Wang et al., 2015 [67]	Examined the survival and proliferation of seed-borne *Listeria monocytogenes* and other members of the seeds microbiota on microgreen plants grown in soil substitute and hydroponic production systems.	During microgreen growth for 10 days, *Listeria monocytogenes* counts on the seed coats increased by 0.7 and 1.3 log, respectively, for soil and hydroponic systems. Similar increases were observed on the edible portion of the microgreens. Seed coats, roots, and cotyledons were most heavily.
3	Di Gioia et al., 2016 [52]	Reported lower microbial populations in recycled fiber mats and on microgreens growing on them than in peat-based mixes and microgreens grown in pure peat.	They suggested that recycled fiber mats may be safer growth media than peat. Recycled textile-fiber and jute-kenaf-fiber may be valid alternatives to peatbecause both ensured a competitive yield, low nitrate content and a similar or higher microbiological quality.
4	Wang and Kniel 2016 [64]	Evaluated the capability of the human norovirus surrogate, murine norovirus (MNV), to internalize from roots to edible tissues of kale and mustard microgreens, as well as virus survival in recirculated water without disinfection.	They found constant high levels of viral RNA in edible tissues. MNV remained infectious in previously contaminated hydroponic systems for up to 12 days and was translocated in edible tissues via roots. Examination of the spatial distribution of bacterial cells on different parts of microgreen plants showed that contaminated seeds led to systematic contamination of whole plants, including both aerial parts and roots.
5	Reed et al., 2018 [68]	Reported that the type of growth medium played an important role in serovar-dependant *Salmonella* survival and growth on microgreens irrigated with contaminated water.	Of the different growth media tested, hydroponic pads resulted in the highest percentage of *Salmonella*-positive samples and the highest *Salmonella* population level on microgreens.

**Table 4:** Scale of attack intensity rating.

Note for Attack Intensity	Surface Attacked
1	If the attack is not observed
2	When the attack is incipient, with less obvious symptoms
3	If the stains occupy up to 5% of the surface
4	When the stains cover between 5–15% of the surface
5	When the stains cover between 15–25% of the surface
6	When the stains cover between 25–40% of the surface
7	When the stains cover between 40–50% of the surface
8	When the stains cover between 50–75% of the surface
9	When the stains cover between 75–100% of the surface

**Table 5:** Studies showing the possibility of influencing the quality and quantity of microgreens with the help of artificial lighting systems.

No.	Reference	Investigation Context	Results
1	Kim et al., 2016 [85]	Reported that there is a potential for LED light in the UV and blue ranges to enhance food safety of hydroponically grown microgreens by treating the water as it circulates.	Light in blue and UV wavelengths is able to kill bacteria. Regardless of the bacterial strain, the sensitivity of illuminated bacterial cells to bile salts and NaCl considerably increased compared to non-illuminated controls.
2	Samuolienė et al., 2016 [86]	Evaluate the role of 638 and 665 nm red light components on quantitative changes in antioxidants and to assess the effect of light quality on the antioxidative status of basil and parsley.	Red spectrum (638 nm) can improve its antioxidant properties, while blue light improves the yield of other phytochemicals related to high-quality products. Increased or supplemental red light significantly increased contents of phenolics, α-tocopherol and ascorbic acid.
3	Lobiuc et al., 2017 [87]	Different ratios of LED blue and red illumination; 4 light treatments were 100% white (White) and various red (R) to blue (B) ratios, as follows: 2R:1B, 1R:1B and 1R:2B, intensities	Growth of microgreens was enhanced with predominantly blue illumination, larger cotyledon area and higher fresh mass. The same treatment elevated chlorophyll a and anthocyanin pigments contents.
4	Zhang et al., 2020 [88]	Effects of light-emitting diode (LED) light on growth, phytochemical compound content and antioxidant capacity, as well as the post-harvest quality of sprouts and microgreens were investigated.	LED light can promote the accumulation of different phytochemicals, such as phenolic compounds, vitamins, glucosinolates, chlorophyll and carotenoids. Meanwhile, the antioxidant capacity could also be significantly increased by growth under LED light, in particular UV-B light. The accumulation of mineral elements (Ca^2+^, Fe^2+^, K^+^) increased after light exposure.
5	Artés-Hernández et al., 2022 [89]	Use of UV and visible spectrum LED lighting to improve the quality of microgreens to enhance their health-promoting compounds.	Illumination with UV and/or different regions of the visible spectrum during growing and shelf life are good abiotic elicitors of the production of phytochemicals in young plants, mainly through the activation of specific photoreceptors.

## Data Availability

Not applicable.
